# Development of a Label-Free Immunosensor for Clusterin Detection as an Alzheimer’s Biomarker

**DOI:** 10.3390/s18010308

**Published:** 2018-01-20

**Authors:** Kamrul Islam, Samar Damiati, Jagriti Sethi, Ahmed Suhail, Genhua Pan

**Affiliations:** 1 Wolfson Nanomaterials & Devices Laboratory, School of Computing, Electronics and Mathematics, Faculty of Science and Engineering, University of Plymouth, Devon PL4 8AA, UK; Jagriti.sethi@plymouth.ac.uk (J.S.); ahmed.suhail@plymouth.ac.uk (A.S.); g.pan@plymouth.ac.uk (G.P.); 2 Department of Biochemistry, Faculty of Science, King Abdulaziz University (KAU), Jeddah 21589, Saudi Arabia

**Keywords:** clusterin, electrochemical biosensor, label-free immunosensor, cyclic voltammetry, square wave voltammetry, screen-printed electrodes, Alzheimer’s disease

## Abstract

Clusterin (CLU) has been associated with the clinical progression of Alzheimer’s disease (AD) and described as a potential AD biomarker in blood plasma. Due to the enormous attention given to cerebrospinal fluid (CSF) biomarkers for the past couple of decades, recently found blood-based AD biomarkers like CLU have not yet been reported for biosensors. Herein, we report the electrochemical detection of CLU for the first time using a screen-printed carbon electrode (SPCE) modified with 1-pyrenebutyric acid *N*-hydroxysuccinimide ester (Pyr-NHS) and decorated with specific anti-CLU antibody fragments. This bifunctional linker molecule contains succinylimide ester to bind protein at one end while its pyrene moiety attaches to the carbon surface by means of π-π stacking. Cyclic voltammetric and square wave voltammetric studies showed the limit of detection down to 1 pg/mL and a linear concentration range of 1–100 pg/mL with good sensitivity. Detection of CLU in spiked human plasma was demonstrated with satisfactory recovery percentages to that of the calibration data. The proposed method facilitates the cost-effective and viable production of label-free point-of-care devices for the clinical diagnosis of AD.

## 1. Introduction

Clusterin (CLU), also known as Apolipoprotein J (Apo J, a highly conserved heterodimeric glycoprotein with molecular weight of 75–80 kDa), has attracted significant scientific attention as it is involved in lipid export during cell differentiation and cell death [1] and can stabilize stressed proteins in a refolding-competent state [2]. It is also implicated in aging and age-related diseases such as neurodegeneration, diabetes, and atherosclerosis [3,4,5], and acts as a biomarker of cellular senescence and oxidative stress [6]. CLU content in the plasma is found to be associated with atrophy of the entorhinal cortex, baseline disease severity, and rapid clinical progression in Alzheimer’s disease (AD) [7]. It is also found in longitudinal brain atrophy in mild cognitive impairment [8]. In addition, CLU gene variants in association with amyloidogenic peptides and CLU contributes to limit amyloid-β (Aβ) species misfolding and facilitates their clearance from the extracellular space [9].

To date, most biomarker studies on AD have focused on the use of cerebrospinal fluid (CSF) and blood plasma levels of β-amyloid, tau, and phosphorylated tau in relation to clinical symptoms and survival as diagnostic specimens [10,11,12,13,14,15]. However, prominent companies and research groups are now realizing that the simplistic amyloid cascade hypothesis that was reasonable to propose 26 years ago is not a reasonable hypothesis any longer [16,17,18]. Besides, AD biomarker tests are either highly invasive (requiring CSF collection) or expensive and labour-intensive (neuroimaging), making them unsuitable for use in primary care, clinical office-based settings, or to assess drug efficacy in clinical trials [19]. Note that the current clinical diagnosis for AD blood test is used to rule out other conditions that may be responsible for mental impairment symptoms in suspected patients, such as thyroid hormones and vitamin B12 levels, not the direct test of AD biomarkers mentioned above. Hence, the development of a rapid and cost-effective means of providing routine screening of elderly patients for AD is of paramount importance. An initial blood-based screening method for AD will be able to reduce the financial burden of AD diagnosis in coming decades by a substantial drop of the need for follow-up referral to expensive neuroimaging or CSF analysis [20,21].

Of the various plasma biomarkers investigated for AD, clusterin has been one that holds promise. CLU is the third most associated late-onset Alzheimer’s disease (LOAD) risk gene according to the AlzGene database [22]. In AD, various studies have shown elevated CLU levels in brain and CSF. Thambisetty et al. (2010) found that CLU is associated with both hippocampal atrophy in AD and mild cognitive impairment (MCI) subjects along with fast progressing, or more aggressive, AD. Evidence from human cerebrospinal fluid, post-mortem brain, and transgenic animal models suggests a plausible link between CLU and AD pathology [7]. To date, the detection of clusterin has been performed by radioimmunoassay, immunohistochemistry, in situ hybridization, Western blotting, and cDNA microarray (Table 1) [23,24,25,26,27,28]. Chung et al. has shown a proof-of-principle, semi-automated, microfluidic point-of-care method of KIM-1 and Cystatin C detection in urine, and proposed that CLU can also be adapted in their method [29]. However, no report has been found for the electrochemical detection of clusterin.

Herein, we report for the first time an electrochemical immunosensor for the detection of CLU. A simple, low-cost biosensor was developed by modifying the carbon electrode with 1-pyrenebutyric acid *N*-hydroxysuccinimide ester (Pyr-NHS) and immobilizing F(ab’)_2_ fragments of CLU antibody to detect CLU antigen specifically. The Pyr-NHS linker utilizes a bifunctional molecule containing succinylimide ester and a pyrene moiety to bind proteins to the carbon surface. Pyrene attaches to the carbon surface by means of the π-π stacking or hydrophobic interactions and does not significantly disturb the electronic structure of the screen-printed carbon working electrode. F(ab’)_2_ fragments of an antibody possess some advantages over whole antibody in terms of close proximity to the surface (within Debye length limit) [30], higher structural stability [31], improved detection limit [32], and additionally reduce non-specific binding from Fc interaction. Wasowicz et al. showed that the Fab’ oriented immobilization for the detection of protein improves the detection limit in comparison to an immunosensor incorporating the whole IgG antibody [32,33]. Jarocka et al. also presented similar results in their study in addition to a comparative study from earlier reports [34,35]. In our study, cyclic voltammetry (CV) and square wave voltammetry (SWV) measurements were used to monitor the generation of each sensing layer and detection efficiency of CLU antigen in the presence of a [Fe(CN)_6_]^3−/4−^ redox system. The obtained results show the successful development of an electrochemical biosensor with good selectivity and sensitivity to detect CLU. Compared to other assays, label-free electrochemical biosensors offer great promise as a robust tool for rapid, sensitive, cost-effective analysis towards a truly disposable tool for screening of AD patients, and can be promoted for point-of-care (POC) application.

## 2. Materials and Methods

### 2.1. Chemicals

Pyr-NHS, phosphate-buffered saline (PBS) tablet, poly(methyl methacrylate) (PMMA), potassium ferrocyanide (K_4_Fe(CN)_6_), potassium ferricyanide (K_3_Fe(CN)_6_), potassium chloride (KCl), ethanolamine, human plasma, and bovine serum albumin (BSA) at biochemical grade were purchased from Sigma Aldrich (Dorset, UK); 100 µg CLU peptide (ab45815) and 1 mL of 0.02 mg/mL complementary anti-CLU (ab39991) in PBS buffer were purchased from Abcam (Cambridge, UK) and the prepared aliquots were stored at −20 °C. Pierce™ Mouse IgG1 Fab and F(ab’)_2_ Micro Preparation Kit and Pierce™ Antibody Clean-up Kit were purchased from Fisher Scientific (Leicestershire, UK).

### 2.2. Screen-Printed Carbon Electrodes (SPCEs)

Screen-printed carbon electrodes (SPCEs) were purchased from Zimmer and Peacock (UK). These electrodes incorporate a conventional three-electrode configuration, printed on ceramic substrates (dimensions: 3.4 × 1.0 × 0.05 cm; length × width × height). Both working (disk-shaped 400 µm diameter) and counter electrodes were made of carbon inks, whereas the reference electrode was made of silver.

### 2.3. Electrochemical Measurments

Electrochemical CV and SWV measurements were performed with a potentiostat (DropSens, Asturias, Spain) and controlled by DropView 8400 1.0 Software. All measurements were carried out at room temperature. All measurements were conducted in a solution of 10 mM K_3_ [Fe(CN)_6_] and 10 mM K_4_[Fe(CN)_6_] containing 100-mM KCl as the supporting electrolyte, and the CV were recorded from −0.2 to 0.5 V at a scan rate of 0.05 V/s without the application of any pre-conditioning potential or accumulation time.

CV is a useful and widely used technique for the characterization of the electrochemical features of electroactive species, but is not always sensitive enough to provide a detailed description of the processes occurring [36]. In this way, SWV was also used for further characterization and to obtain more distinctive electrochemical signatures. Voltammograms were obtained by sweeping the potential from −0.15 V to +0.45 V with a step potential of 5 mV, an amplitude of 25 mV, and at a frequency of 15 Hz.

### 2.4. Preparation and Characterization of Antibody Fragments

#### Preparation of F(ab’)_2_ Fragments

**Figure 1:**
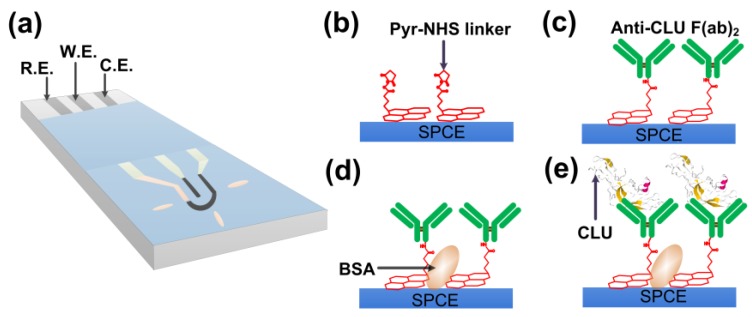
Schematic illustration of the electrochemical detection system. (**a**) The screen-printed carbon electrodes (SPCE) electrode; (**b**–**e**) surface modification with linker, F(ab’)_2_ fragments of CLU antibody (Anti-CLU F(ab’)_2_), bovine serum albumin (BSA), and CLU. Pyr-NHS: 1-pyrenebutyric acid *N*-hydroxysuccinimide ester.

Antibody fragments digested by ficin were used instead of whole antibody to avoid random orientation of the antigen binding site toward the sensor surface leading to monolayer [37,38]. This prevents loss of the biological activity of the antibody and displays a highly-controlled orientation that is expected to maximize their antigen-binding efficiency with a concomitant increase in sensitivity and selectivity (Figure 1c) [39,40,41,42].

F(ab’)_2_ fragments of CLU antibody (Anti-CLU F(ab’)_2_) were prepared by using Pierce™ Mouse IgG1 Fab and F(ab’)_2_ Micro Preparation Kit. In short, a Pierce™ Antibody Clean-up Kit was used to remove 0.5% added BSA from antibody solution. Immobilized ficin was equilibrated by swirling the vial to obtain an even suspension. A wide bore pipette tip was used to place the 200 µL of the 33% slurry into the 0.8 L spin column, which was placed in a 2 mL microcentrifuge tube, followed by centrifugation at 5000 × *g* for one minute. The pellet was washed with 0.5 mL of digestion buffer, followed by centrifugation again at the same conditions. IgG sample was prepared in a Zeba Spin Desalting Column with 100 µL of antibody sample by using a digestion buffer and centrifugation as instructed on the kit. The fragments were generated by adding prepared IgG sample to the spin column tube containing the equilibrated immobilized ficin. Digestion reaction was done by incubation for 24–30 h for the generation of F(ab’)_2_ fragments with a table-top rocker at 37 °C. Samples were then washed and purified according to the instructions of the preparation kit. Finally, antibody fragments were characterized on Criterion XT 4%–12% Bis-Tris precast gradient gels according to the outlines of the manufacturer (Bio-Rad, Hemel Hempstead, UK). Aliquots were diluted with 20 µL Laemmli Sample buffer for the precast gel slots. Gels were run at 120 V constant, 0.09 A max for 2 h in a BioRad Mini Protean II system, stained for Gel Code Blue (Pierce) and analysed using Image J software to determine protein band intensities.

### 2.5. Surface Modification of SPCE Electrode

Screen-printed carbon electrodes were exposed to 2 mM Pyr-NHS for 4 h at 4 °C, followed by methanol rinsing to remove free Pyr-NHS molecules. F(ab’)_2_ fragmented Anti-CLU antibodies (20 µg/mL) in PBS (pH 7.4) were immobilized on the SPCE for 4 h at 4 °C and then rinsed with PBS to remove unbound antibodies. Subsequently, 0.5% BSA in PBS was added and incubated for 4 h at 4 °C. After rinsing with PBS, the modified electrodes were incubated with different concentrations of CLU at 37 °C for ~60 min. Essentially, the modification of the electrode surface requires several hours, but it can be pre-prepared and stored at 4 °C. The detection of a single antigen concentration requires incubation at 37 °C for 1 h, while SWV measurement takes approximately 3–5 min.

## 3. Results and Discussion

### 3.1. Characterization Using Non-Reducing Electrophoresis (SDS-PAGE)

#### Characterization of F(ab’)_2_ Fragments

**Figure 2:**
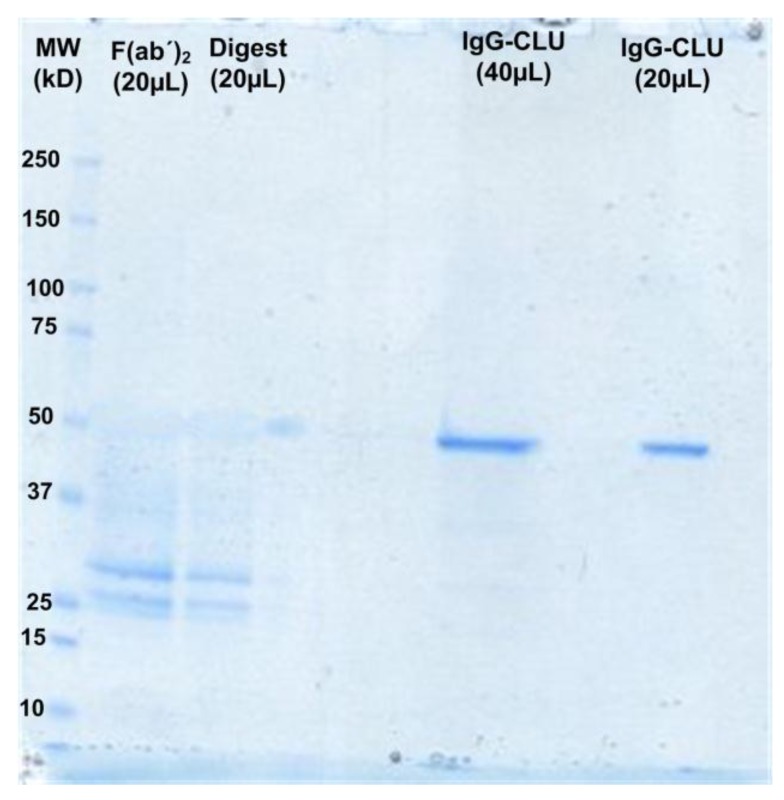
SDS-PAGE analysis (12% gel; non-reducing conditions) of the full-length CLU antibody and their F(ab’)_2_ fragments: (column 1 is molecular weight marker; column 2 is F(ab’)_2_ fragments of CLU antibody; column 3 is digest fragments; and columns 4–5 are full-length Anti-CLU IgG.

Figure 2 illustrates the SDS-PAGE (12%) analysis of the full-length CLU antibodies and their F(ab’)_2_ fragments under the non-reducing conditions. The column for F(ab’)_2_ in Figure 2 clearly shows one major band around 29 kDa, indicating that a significant amount of F(ab’)_2_ was produced. Both columns of IgG-CLU present major bands around 50 kDa, which can be attributed to the prepared whole CLU antibody. Column 3 shows the digest portion that contains Fab with Fc. A faint band is visible around 30 kDa in both F(ab’)_2_ and digest columns, indicating that some Fab was produced due to the reduction of F(ab’)_2_ during the enzymatic fragmentation process, which is expected according to the data presented in the preparation kit manual.

### 3.2. Electrochemical Characterization of the Modified Electrode

**Figure 3:**
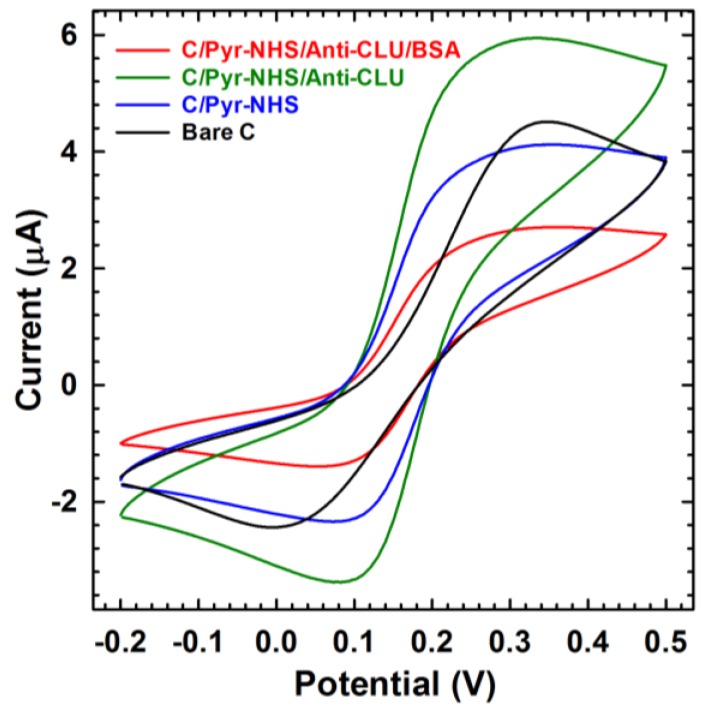
Cyclic voltammetry (CV) has been performed with 10 mM [Fe(CN)_6_]^3−/4−^ system (1:1) and 100 mM KCl at 0.05 V/s scan rate at bare carbon, Pyr-NHS linker, Anti-CLU F(ab’)_2_, and BSA-coated SPC electrodes.

Electrochemical biosensors have been widely reported for the detection of dementia because of their inherent sensitivity [43]. Therefore, CV was performed (and the response current vs. applied potential plotted) to confirm the changes in the electrochemical properties after each electrode modification step (Figure 3). Upon the self-assembly of the linkers on the surface of the SPCE electrode, the peak current decreased from 4.40 to 3.76 µA (15%), reflecting an increase in the electron transfer resistance. Subsequently, after the immobilization of the anti-CLU antibody, electron transfer significantly increased (47%). This may be attributed to the available non-binding sites (i.e., free NH^3+^ group) on the Anti-CLU F(ab’)_2_ immobilized SPCE that play an important role, resulting in accelerated electron transfer between Anti-CLU F(ab’)_2_ and the SPCE. Note, that lysine contains a primary amine (-NH_2_) in the side chain as an antibody functional group, also called epsilon-amine. Owing to the positive charge of epsilon-amine at physiologic conditions, primary amines are usually outward facing (i.e., on the outer surface) on proteins; thus, they are usually accessible for conjugation without denaturing the protein structure. The epsilon-amines act as an electron donating group, leading to *n*-doping on the SPCE surface. However, the magnitude of the current response decreases after the immobilization of BSA due to blocking of the non-specific binding sites of Anti-CLU F(ab’)_2_ that hinder the electron transfer between the medium and electrode, indicating the immobilization of BSA onto the Anti-CLU F(ab’)_2_/Pyr-NHS/SPCE bioelectrode [44].

### 3.3. Scan Rate Dependence of Peak Current

**Figure 4:**
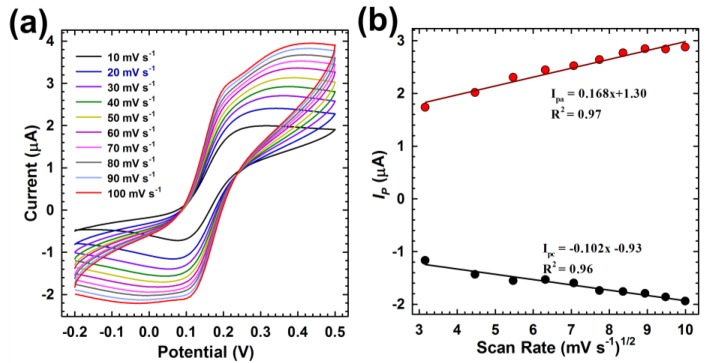
(**a**) Cyclic voltammograms of C/Pyr-NHS/Anti-CLU F(ab’)2/BSA in [Fe(CN)_6_]^3−/4−^ solution containing KCl with a scan rate from 10 to 100 mV/s; (**b**) dependence of the redox peak currents on the scan rates.

After performing the evaluation of the generated sensing layers, we also conducted sensing experiments on the modified electrodes to characterize the redox process that is taking place on C/Pyr-NHS/Anti-CLU-F(ab’)_2_/BSA modified electrodes. This study has been conducted in [Fe(CN)_6_]^3−/4−^ solution containing KCl with a scan rate from 10 to 100 mV/s (Figure 4a). With increase in the scan rate, there was an increase in both the cathodic and anodic peak currents, accompanied by a small shift and increased peak-to-peak separation. The linearity dependence is also confirmed by the regression coefficient (R^2^) value (R^2^ = 0.91 for *I_pa_* and R^2^ = 0.96 for *I_pc_*), and is indicative of a surface-controlled diffusion and quasi-reversible process. This reveals that the electron transfer between [Fe(CN)_6_]^3−/4−^ solution and electrode could be easily performed, and it was a surface-confined electrochemical process. The values of the slope, intercept and correlation coefficient are given in Figure 4b. The diffusion co-efficient (*D*) value of the redox species from the electrolyte to the C/Pyr-NHS/Anti-CLU-F(ab’)_2_/BSA immunoelectrode was calculated using the Randles–Sevcik equation [44]:

$$I_{p}=(2.69\times 10^{5})n^{3/2}AD^{1/2}Cv^{1/2}$$

where *I*_p_ is the peak current of the immunoelectrode (*I*_pa_ anodic and *I*_pc_ cathodic), *n* is the number of electrons involved or electron stoichiometry (1), *A* is the surface area of the immunoelectrode (1.26 × 10^−3^ cm^2^), *D* is the diffusion co-efficient. *C* is the concentration of redox species (10 mM [Fe(CN)_6_]^3−/4−^), and *v* is the scan rate (50 mVs^−1^). The *D* value has been obtained to be 1.11 × 10^−5^ cm^2^s^−1^.

The surface concentration of the C/Pyr-NHS/Anti-CLU-F(ab’)_2_/BSA immunoelectrode can be estimated from the plot of current versus potential (*C_V_*) using the Brown–Anson model [44], via the following equation:

$$I_{p}=\frac{n^{2}F^{2}\gamma Av}{4RT}$$

where *n* is the number of electrons transferred (1), *F* is the Faraday constant (96,485.34 C mol^−1^), *γ* is the surface concentration of the corresponding electrode (mol cm^−2^), *A* is the surface area of the electrode (1.26 × 10^−3^ cm^2^), *v* is the scan rate (50 mVs^−1^), *R* is the gas constant (8.314 J mol^−1^ K^−1^), and *T* is room temperature (25 °C). The surface concentration of C/Pyr-NHS/Anti-CLU-F(ab’)_2_ (8.81 × 10^−8^ mol cm^−2^) was higher than that of the SPCE (6.41 × 10^−8^ mol cm^−2^) electrode.

The value of the heterogeneous electron transfer rate constant (*K*_s_) obtained for the C/Pyr-NHS/Anti-CLU-F(ab’)_2_/BSA immunoelectrode was calculated from the Lavrion model [45]:

$$K_{s}=\frac{mnFv}{4RT}$$

where *m* is the peak-to-peak separation (0.25 V), *n* is the number of transferred electrons (2), *F* is the Faraday constant (96,485.34 C mol^−1^), *v* is the scan rate (50 mVs^−1^), *R* is the gas constant (8.314 J mol^−1^ K^−1^), and *T* is room temperature (25 °C). The value of *K*_s_ was found to be 0.24 s^−1^ for *m* = 0.25 V, which indicates fast electron transfer between the immobilized antibodies and electrode at the SPCE interface.

### 3.4. Quantitative Detection of CLU Using Electrochemical Measurement

**Figure 5:**
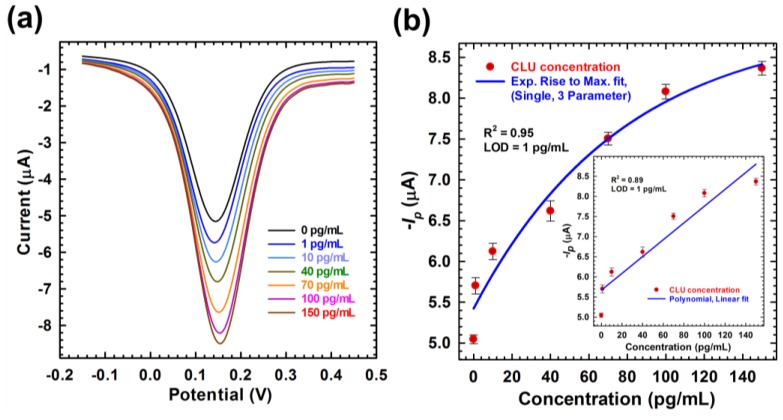
(**a**) SWV peaks of the C/Pyr-NHS/Anti-CLU F(ab’)_2_/BSA/CLU-modified electrode in the presence of different concentrations of CLU (0–150 pg/mL); (**b**) Exponential rise to maximum fit curve with regression analysis and limit of detection (LOD). Inset: linear fit curve with regression analysis and LOD.

The electrochemical response studies (Figure 5a) of the C/Pyr-NHS/Anti-CLU F(ab’)_2_/BSA immunoelectrode have been performed as a function of CLU concentration (0~150 pg mL^−1^) in 10 mM [Fe(CN)_6_]^3−/4−^ solution containing 100 mM KCl using the SWV technique by sweeping the potential from −0.15 V to +0.45 V with a step potential of 5 mV, an amplitude of 25 mV, and at a frequency of 15 Hz. Here, SWV measurement was performed for BSA/Ab/Pyr NHS/C surface at 0 pg mL^−1^CLU as a control experiment. The peak values were obtained through DropView 8400 1.0 Software, which showed the decrease of the peak current intensities with the increase of CLU concentration. The signal reduction was caused by the blockage of the surface, making the electron transfer process between the C/Pyr-NHS/Anti-CLU F(ab’)_2_ surface and [Fe(CN)_6_]^3−/4−^ system more difficult. The peak value was around 0.14 V, corresponding to the biochemical reaction between Anti-CLU F(ab’)_2_ and CLU. The data were fitted to a single three-parameter exponential raise to maximum equation $\left(y=y_{0}+a\left(1-e^{-bx}\right)\right)$ using SigmaPlot 13.0 software (solid blue line in Figure 5b), where *y* is the accumulated current response (µA), *y*_0_ is the background current or current at saturation (µA), *a* is the initial current response (µA), *b* is the kinetic constant (slope of the fitting curve—regarded here as sensitivity), and *x* is the CLU concentration (pg mL^−1^). The fabricated electrochemical sensor exhibited a good detection range 1—100 pg mL^−1^ toward CLU with nonlinear regression co-efficient of 0.952 (Figure 5b). A linear fit is also shown in the inset of Figure 5b. The sensitivity of the fabricated immunoelectrode calculated from the slope of the fitting curve was found to be 0.013 µA pg^−1^ mL^−1^. The sensitivity was lower, as the NH_3_ of the CLU will donate electron charge at the interface with SPCE electrode. This will reduce the p-doping level of the electrode [46]. The addition of a high concentration of CLU (above 1 ng mL^−1^) had an impact on the SWV signals response due to the saturation of sensor surface (data not shown). A limit of detection of 1 pg mL^−1^ was calculated for the biosensor following the method described by Armbruster et al. [47].

### 3.5. Selectivity and Validity of the Sensor

**Figure 6:**
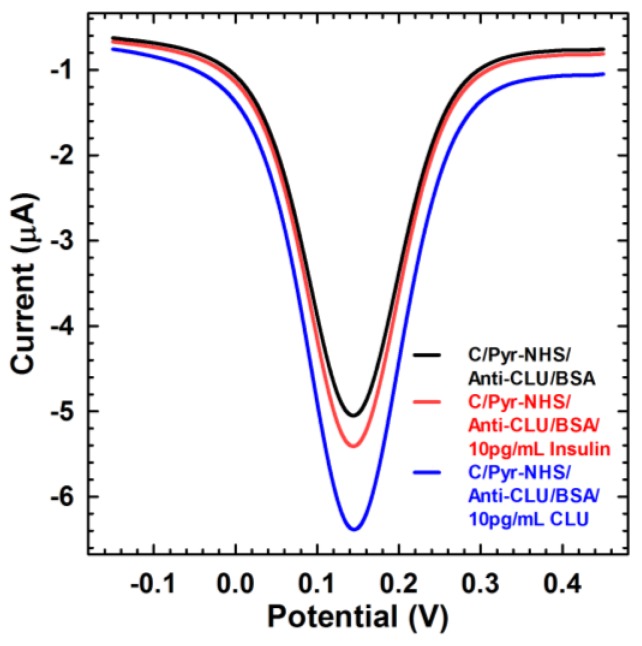
SWV of negative control experiment with CLU and Insulin.

To test the specificity to detect CLU, the developed sensor was incubated with 10 pg mL^−1^ insulin and the obtained SWV signal was compared with CLU at the same concentration (Figure 6). The SWV signal did not change significantly after the addition of insulin. The small shift in the signal may be attributed to unspecific adsorption on the sensor surface. In contrast, a noticeable response was achieved after addition of CLU due to the formation of an antibody–antigen complex. These results confirm the selectivity of the fabricated biosensor to detect CLU protein.

To test the analytical applicability of the proposed method, human plasma sample (reconstituted from lyophilized plasma, Sigma-Aldrich p9523, Dorset, UK) serving as blank was spiked with CLU antigen in two different concentrations: 10 pg mL^−1^ and 100 pg mL^−1^. This was performed in triplicate, and only by following SWV data. The recoveries obtained were 62.60% and 77.70%, respectively, to that of the calibration data (Table 2). For pg mL^−1^ concentration level, these recovery percentages are expected [48]. Overall, the results were found accurate in close-to-real conditions and validate the analytical applicability of the proposed method.

## 4. Conclusions

The present work reports a proof-of-principle electrochemical immunosensor for the detection of CLU on SPCEs for the first time. This platform—which is based on modified screen-printed electrodes—is a low-cost and robust detection platform. The immunosensor shows broad detection range (1~100 pg mL^−1^), high sensitivity (0.013 µA pg^−1^ mL^−1^), and selectivity to CLU. Additionally, the detection limit (1 pg mL^−1^) is very low compared to the average concentration of CLU found in AD patients (~2.50 µg mL^−1^ according to ADNI database). The detection of protein in spiked plasma was demonstrated, and satisfactory recovery percentages of 62.60% and 77.70% for 10 pg mL^−1^ and 100 pg mL^−1^ concentrations were obtained, respectively, to that of the calibration data. Related to AD biomarkers in blood, the possibility of measuring a group or panel of different proteins is a desirable objective to provide faster and more accurate diagnostics. Simultaneous detection of the possible AD biomarkers in blood such as complement factor H, α-2-macroglobuline, apolipoprotein E, pancreatic polypeptide, and clusterin would be necessary on a multiplexed electrochemical immunosensor platform. However, additional efforts are necessary for the simultaneous detection of biomarkers, since each protein type might have high differences in threshold levels and would vary in their detection sensitivity levels [49]. Along with this electrochemical system, paper-based and amperometry-based microfluidic methods would provide the advantages of working on small volumes, with short assay period, high sensitivity, and accelerated kinetic processes [50,51,52]. Finally, the obtained results should facilitate the application of these antibody-functionalized SPCE surface modifications for biosensing and the development of a promising platform for clinical diagnosis in the future.

## Figures and Tables

**Table 1:** Detection of clusterin (CLU) by various methods. IHC: immunohistochemistry; ISH: in situ hybridization; WB: Western blotting; cDNA-MA: cDNA microarray; NB: Northern blotting; LN: lymphoid neoplasms; ALCL: anaplastic large-cell lymphoma.

Detection Method	Organ	Disease	Reference
Radioimmunoassay	Kidney, urine	Nephrotoxicity	[23]
IHC, ISH	Breast	Cancer	[24]
IHC, WB	LN	ALCL	[25]
IHC, cDNA-MA, WB/NB	Liver	Cancer	[26]
RT-PCR, WB, IHC, ISH	Kidney, urine	Nephrotoxicity	[27]
IHC, ISH	Kidney, blood, urine	Nephrotoxicity	[28]

**Table 2:** Determination of CLU in spiked plasma samples (*n* = 3).

Plasma Sample	CLU Spiked (pg mL^−1^)	CLU Found (pg mL^−1^)	Recovery (%)
S#1	10	8.07, 6.17, 4.54	62.60
S#2	100	86.70, 77.85, 68.55	77.70
